# Revised fission yeast gene and allele nomenclature guidelines for machine readability

**DOI:** 10.1093/genetics/iyad143

**Published:** 2023-09-27

**Authors:** Manuel Lera-Ramírez, Jürg Bähler, Juan Mata, Kim Rutherford, Charles S Hoffman, Sarah Lambert, Snezhana Oliferenko, Sophie G Martin, Kathleen L Gould, Li-Lin Du, Sarah A Sabatinos, Susan L Forsburg, Olaf Nielsen, Paul Nurse, Valerie Wood

**Affiliations:** University College London, Department of Genetics Evolution and Environment, Darwin Building, 99-105 Gower Street, London WC1E 6BT, UK; University College London, Department of Genetics Evolution and Environment, Darwin Building, 99-105 Gower Street, London WC1E 6BT, UK; University of Cambridge, Department of Biochemistry, Cambridge CB2 1GA, UK; University of Cambridge, Department of Biochemistry, Cambridge CB2 1GA, UK; Boston College, Department of Biology, Chestnut Hill, MA 02467, USA; Institut Curie, Université Paris-Saclay, CNRS UMR3348, Orsay 91400, France; The Francis Crick Institute, London NW1 1AT, UK; Randall Centre for Cell and Molecular Biophysics, School of Basic and Medical Biosciences, King’s College London, London SE1 1UL, UK; University of Geneva, Department of Molecular and Cellular Biology, Geneva 1211, Switzerland; Vanderbilt University School of Medicine, Department of Cell and Developmental Biology, Nashville, TN 37232, USA; National Institute of Biological Sciences, Beijing 102206, China; Toronto Metropolitan University, Department of Chemistry & Biology, Toronto M5B 2K3, Canada; Molecular and Computational Biology Program, University of Southern California, Los Angeles, CA 90089, USA; Department of Biology, Cell cycle and genome stability Group, University of Copenhagen, Copenhagen N DK2100, Denmark; The Francis Crick Institute, London NW1 1AT, UK; University of Cambridge, Department of Biochemistry, Cambridge CB2 1GA, UK

**Keywords:** nomenclature, allele, genotype, standardization, FAIR, model organism, fission yeast, database, gene variant, knowledge base

## Abstract

Standardized nomenclature for genes, gene products, and isoforms is crucial to prevent ambiguity and enable clear communication of scientific data, facilitating efficient biocuration and data sharing. Standardized genotype nomenclature, which describes alleles present in a specific strain that differ from those in the wild-type reference strain, is equally essential to maximize research impact and ensure that results linking genotypes to phenotypes are Findable, Accessible, Interoperable, and Reusable (FAIR). In this publication, we extend the fission yeast clade gene nomenclature guidelines to support the curation efforts at PomBase (www.pombase.org), the *Schizosaccharomyces pombe* Model Organism Database. This update introduces nomenclature guidelines for noncoding RNA genes, following those set forth by the Human Genome Organisation Gene Nomenclature Committee. Additionally, we provide a significant update to the allele and genotype nomenclature guidelines originally published in 1987, to standardize the diverse range of genetic modifications enabled by the fission yeast genetic toolbox. These updated guidelines reflect a community consensus between numerous fission yeast researchers. Adoption of these rules will improve consistency in gene and genotype nomenclature, and facilitate machine-readability and automated entity recognition of fission yeast genes and alleles in publications or datasets. In conclusion, our updated guidelines provide a valuable resource for the fission yeast research community, promoting consistency, clarity, and FAIRness in genetic data sharing and interpretation.

## Introduction

The fission yeast *Schizosaccharomyces pombe* is a prominent model organism for the understanding of molecular mechanisms underlying fundamental cellular processes (Hayles and Nurse 2017). Only 5 species are currently recognized in the *Schizosaccharomyces* clade (*S. pombe, S. japonicus, S. octosporus, S. cryophilus,* and *S. osmophilus*), and the genomes of all 5 species have been sequenced (Wood *et al*. 2002; Rhind *et al*. 2011; Jia *et al*. 2023). All have been the subject of evolutionary studies and *S. japonicu*s is also emerging as a powerful model for evolutionary cell biology (Russell *et al*. 2017; Oliferenko 2018; Rutherford *et al*. 2021).

Standardized nomenclature for the genes, gene products, and isoforms of a model species is important for clear communication of scientific data, biocuration, data sharing, and recording the provenance of genetic entities. Unambiguous nomenclature syntax becomes paramount as databases and journals attempt to identify named entities using lexical or rule-based methods to establish links between journals and databases (Raciti *et al*. 2018), or to automate the first steps in the curation process, such as the detection of new variants (Davis *et al*. 2022). Accurate entity recognition is also a necessary initial step in data mining and machine learning over biological knowledge (Perera *et al*. 2020).

Standardized nomenclature is key for the “FAIRification” (Findable, Accessible, Interoperable, and Reusable) (Wilkinson *et al*. 2016) of results and biological resources produced in model organism research. A fission yeast publication often uses tens of engineered strains carrying targeted mutations, tags, regulating sequences, engineered sensors, etc. Because of inconsistencies, it is currently not possible to parse genotypes in published strain lists. Genotype parsing would greatly facilitate the annotation of genotype-to-phenotype relationships and would allow model organism databases to provide inventories of published strains that are queryable by their allele features, greatly increasing the reusability of resources and preventing redundancy in research. Standardized nomenclature will also serve to (i) detect errors (e.g. alleles referring to residues not present at the indicated position) and prevent their incorporation into knowledge bases, (ii) harmonize inconsistencies and typographical errors in existing and new datasets, and (iii) identify synonymous alleles with different names (manuscript in preparation, M. L.-R. *et al*).

### Gene nomenclature standardization in fission yeast

The fission yeast genome sequencing projects assigned gene systematic identifiers (locus_tag in the International Nucleotide Sequence Database Collaboration -INDSC- databases) for known or predicted gene products in each species. Identifiers start with SP for *S. pombe*, SJAG for *S. japonicus* and are used to refer to genes precharacterization, or in high throughput datasets. Unique identifiers are minted by model organism databases when new genes are discovered. In addition, genes that have been published are given a “gene symbol” by authors (e.g. SPAPB1A10.09 is the systematic identifier, and *ase1* is the gene symbol). Consequently, all known fission yeast genes have a systematic identifier, but only those published or with clear orthology to a named gene in another species have a gene symbol. In this manuscript, “naming a gene” means assigning a symbol to a gene. The term “gene name” is equivalent to “gene symbol” in yeast, but it is used differently in other species. For example, the human gene with the symbol PRC1 has a gene name “protein regulator of cytokinesis 1”. Consequently, we have used the species-agostic term “gene symbol” hereon. Fission yeast gene symbols are 3 lowercase Latin letters plus one or more digits (e.g. *cdc25*). In publications, they are printed in lowercase italics when referring to the gene (*cdc25*), and not italicized with the first letter capitalized when referring to the gene product (e.g. Cdc25). Historically a “p” was sometimes added to designate the gene product (e.g. Cdc25p).

The naming system for protein-coding genes in the fission yeast *Schizosaccharomyces pombe* is well-established. However, minor extensions and further systemization are required to provide a scalable way to name long noncoding RNA genes.

### History and conflicts of current gene naming system

Before genome sequencing, genes were named after characterized mutant alleles. The 3-letter gene symbols were usually an acronym or diminutive of the phenotype at a specific locus (i.e. *cdc* for cell division cycle mutant, *rad* for radiation sensitivity). Inevitably this practice caused inconsistencies when the same symbol was given to different loci in budding and fission yeast. A notable example is the gene coding for the major cyclin-dependent kinase, which is *cdc2* in *S. pombe* and *CDC28* in *S. cerevisiae*. Conversely, *S. pombe cdc28* and *S. cerevisiae CDC2* also exist and are different genes. In such cases, authors usually mention both orthologs or use a shared synonym to avoid confusion. For example, “*cdc2/CDC28*” or the common synonym “*cdk1*”, which also matches the human symbol. Because of the volume of publications that refer to these genes by their current symbols, these symbols will not be changed.

Post genome sequencing, the fission yeast Gene Nomenclature Committee (GNC) was convened in 2002 by Paul Nurse, Mitsuhiro Yanagida, and Amar Klar and has since served to accept symbol reservations, ensure uniqueness and arbitrate symbol conflicts if required (see https://www.pombase.org/submit-data/gene-names). Uncharacterized *S. pombe* genes have been named after their budding yeast orthologs to reduce nomenclature divergence between species. To reduce discrepancies further, *S. pombe* loci have occasionally been renamed to match *S. cerevisiae* loci, especially when the *S. cerevisiae* symbol had been adopted across species and was used also by fission yeast researchers. For example, *rad51* in *S. pombe* was formerly named *rhp51* (for “*rad51* homolog in *S. pombe*”), but named *rad51* in most eukaryotes, (*rhp51* remains a synonym).

### Genotypes and alleles in the context of strain lists

A model organism strain is characterized by its genotype, the alleles present in that strain that differ from those in the wild-type reference strain. The allele features described in a genotype can be separated into 2 main categories:

- **Gene variant features** are described using the wild-type nucleotide or amino acid sequence as a reference and include all substitutions and deletions. Insertions that do not introduce new functional sequence features are also included. Gene variant features are not restricted to gene products and can also affect regulatory elements, such as promoters.
- **Nonvariant features** specify the insertion locus and describe exogenous functional sequence elements (tags, promoters, terminators) or special alleles such as chimeras, gene fusions, or sensors.

For instance, the insertion of amino acids PKKKRKV, a nuclear localization signal (NLS), at the N-terminal of a protein would not be considered a gene variant feature, because it introduces a new functional element. However, inserting the same number of amino acids to disrupt the function of a domain of the protein would be considered a gene variant feature. Ultimately, this distinction is only used to favor NLS-ase1 as an allele name over ase1-M1-PKKKRKV, even though both are correct (see below for nomenclature rules). Additionally, alleles in a strain genotype can have either or both of these elements. For example, *cdc25-C480S* has only gene variant features, *cdc25-GFP* has only nonvariant features and *cdc25-C480S-GFP* has both. FlyBase has defined relationships between nonvariant features present in engineered alleles and the allele itself that categorize the different types of nonvariant features (Gramates *et al*. 2022):

- **Tagged with**: features that lead to translational or transcriptional fusion of the gene product with an exogenous sequence motif (purification or fluorescent tag, protein or RNA localization motif, degron tag, etc.).
- **Regulatory region**: engineered regulatory region used to drive expression of a gene product (promoter, terminator, etc.).
- **Also carries**: anything that is not fused to the gene product or is not aimed at affecting its expression, such as selection markers, recombination sites, and transposon sequences.
- **Encoded tool**: entire gene products that are not conventional variants. These can be chimeras, gene fusions, sensors, adaptors (like TIR1 for the degron (Nishimura *et al*. 2009)), etc. The encoded tool can also be tagged (Lifeact-GFP (Riedl *et al*. 2008)), regulated (GFP reporter under the control of the promoter of a gene of interest) or carry extra sequences such as selection markers.

### Genotypes and alleles in the context of genotype–phenotype relationships

In genotype-to-phenotype relationships, the genotype of a strain is defined with respect to the control strain of a particular experiment. For example, if the localization of fluorescently tagged protein X is abnormal when deleting gene Y, the genotype–phenotype relationship is that genotype YΔ leads to abnormal localization of protein X. In other words, the fluorescently labeled protein is not part of the genotype because it is present in both control and mutant strains. This is not only restricted to tags and applies when using a mutant background in the experiment design, e.g. to cause a cell cycle arrest. In the fission yeast databases (PomBase (Harris *et al*. 2022) and JaponicusDB (Rutherford *et al*. 2021)), referred to as PomBase hereon, this information can be captured as “background”, and is not part of the genotype in phenotype annotations. Consequently, phenotype annotations are mostly assigned to gene variant features. There are some exceptions to this convention, such as annotations to indicate that tagging a gene product alters its function, whether that is intended (NLS) or accidental (GFP).

### Limitations of genotype–phenotype annotations in PomBase and JaponicusDB

PomBase does not capture regulatory regions that impact gene expression as part of the genotype. The change in expression level (e.g. knockdown/overexpression) is recorded instead since gene expression changes driven by promoters are often conditional and not intrinsic, unlike changes in gene product sequence. The assumption is that achieving the same level of expression with a different promoter would have the same effect. Furthermore, certain simplifications are made when describing genotypes. For example, PomBase does not capture the difference between strains expressing an allele from a plasmid or the native locus in phenotype annotations. Additionally, independently generated gene deletions are treated as the same allele, even though subtle differences may exist due to the targeting strategy or recombination event used (S. Sabatinos, pers. comm.). It is possible to assign a different name to a deletion allele to distinguish it from other deletion alleles, but PomBase does not track this information by default. However, all phenotype annotations are linked to publications, so the exact alleles can be identified.

### Allele naming conventions for fission yeast

The only published nomenclature conventions for *S. pombe* alleles were compiled by Jürg Köhli in the early days of genetic engineering and have served the community well for over three decades (Kohli 1987). These rules cover the designation of alleles generated by forward genetics as well as engineered gene disruptions. Historically, *S. pombe* alleles identified by forward genetics were printed in italics and designated by a combination of letters and numbers following the gene symbol as a superscript (*cdc2^ts^*), or separated by a hyphen (*arg1-230*, *ade6-M210*, following Köhli's rules) (Kohli 1987). However, superscripts are deprecated (see below).

The *S. pombe* genetic toolbox has greatly developed since then, and allele naming practices have been organically extended by the community. In general, naming conventions have converged but systematization is required for variants affecting particular sequence domains such as the RNA polymerase carboxy-terminal domain (CTD) tandem repeats (Karagiannis and Balasubramanian 2007), engineered alleles containing functional sequence domains such as promoters and terminators, and complex constructs such as chimeras.

### Summary

Here we present the updated gene and allele nomenclature guidelines for the fission yeast clade genes and alleles. We provide examples and explanations of how to apply these rules to different types of genetic modifications, such as insertions and deletions. This nomenclature system will facilitate systematization, improve data sharing, and ensure the accuracy and traceability of allele information to facilitate machine readability and automated entity recognition of fission yeast genes and alleles in publications and datasets.

## Gene naming rules

### General/global rules for gene symbol assignment

- Gene symbols must be unique within the species.
- Symbols should contain 3 Latin letters followed by one or more digits and no punctuation. Some historical exceptions to this rule are:
  ○ Mitochondrial ribosomal proteins (e.g. *mrpl1, mrpl66, etc.*), since their yeast homologs also use this 4-letter prefix “mrpl”.
  ○ Cytoplasmic ribosomal proteins *rpl35A01, rpl35A02* to resolve a conflict between rpl35 and rpl35A.
  ○ *pabp* to avoid confusion between poly(A)-binding protein and pab1 phosphatase.
  ○ *cox1-I1b*, *cox1-I2b* (intronic cox1 protein-coding transcripts)
  ○ Mating type locus genes: *mat1-Mc*, *mat1-Mi*, etc.
  ○ Nucleolar RNAs: *snR51b*, *snoR02*, *snoU14*, *snoZ*, *snoZ16* etc.
  ○ Previously published noncoding RNAs: see next section.
- Symbols must not contain references to a species, i.e. sp or p for *S. pombe*.
- Existing 3-letter codes should not be used with a different meaning in new genes, e.g. *cdc* should always refer to “cell division cycle”.

### A note on tRNA genes

Most tRNAs in fission yeast do not have gene symbols, and any existing symbols come from forward genetic screens (*spl1* SeP-Like, *scn1*, and *scn2* Suppressor of Cut Nine, etc.). We recommend using the systematic identifiers (see above) to refer to tRNAs. In the text of publications, they can be described “nuclear Alanine tRNA 1(SPATRNAALA.01)”, and “mitochondrial Alanine tRNA 1 (SPMITTRNAALA.01)”, since the numerical suffixes in the systematic identifiers are unique for each nuclear or mitochondrial amino acid family.

### New rules for ncRNA naming

In the current *S. pombe* genome annotation, there are a total of 12,683 genes, of which 7,518 are ncRNAs. To avoid depleting the 3-letter code pool, and because the functions of many ncRNAs are to regulate the expression of other coding genes, the fission yeast GNC has adapted the guidelines of the Human Genome Organisation Gene Nomenclature Committee (HGNC, www.genenames.org) (Seal *et al*. 2020) when naming ncRNA genes (summarized in Table 1):

- [regulated gene symbol]-ot[index] (**O**verlapping **T**ranscript forward strand) for ncRNA genes that overlap a protein-coding gene on the same strand. Example: *ase1-ot1*, *ase1-ot2*.
- [regulated gene symbol]-as[index] (overlapping **A**nti**S**ense) for ncRNA genes that overlap a protein-coding gene on the opposite strand. Example: *ase1-as1*, *ase1-as2*.
- [regulated gene symbol]-dt (**D**ivergent **T**ranscript) for ncRNA genes that do not overlap with a protein coding gene, but are divergently transcribed (they share a bidirectional promoter). Example: *ase1-dt*.
- [regulated gene symbol]-it[index] (**I**n**T**ronic) for ncRNA genes contained within an intron of a protein-coding gene on the same strand. Example: *ase1-it1*
- Intergenic noncoding RNAs can be assigned conventional gene symbols (3-letter code plus digits) when functionally characterized as they likely have functions unrelated to regulating gene expression of an adjacent protein-coding gene. Example: *aal1 (SPNCRNA.1530)*

**Table 1.** Noncoding RNA nomenclature rules, adapted from HGNC rules (Seal *et al*. 2020).

Symbol pattern	Use case	Example
[regulated gene symbol]-ot[index]	ncRNA genes that overlap with a protein-coding gene and are on the same strand.	*ase1-ot1*, *ase1-ot2*
[regulated gene symbol]-as[index]	ncRNA genes that overlap with protein-coding gene and are on the opposite strand	*ase1-as1, ase1-as2*
[regulated gene symbol]-dt	ncRNA genes that do not overlap with a protein-coding gene, but are divergently transcribed	*ase1-dt*
[regulated gene symbol]-it[index]	For ncRNA genes contained in the introns of a protein-coding gene, on the same strand	*ase1-it1, ase1-it2*
Three-letter code plus digits	Intergenic noncoding RNAs	*aal1(SPNCRNA.1530)*

These rules will be applied moving forward, but existing exceptions (*rnpB*, IRC1-L, *nc-pho1, nc-tco1, nc-tgp1, cti6-antisense-1*, *lncRNA584,* etc.) will be exempted.

## Genotype description rules

Below we provide guidelines to describe fission yeast genotypes in strain lists, extending the initial set of rules from (Kohli 1987). These guidelines capture conventions that have organically emerged or support the most common representation where more than one has been used in the literature. After discussions and approval by a diverse representation of the fission yeast community, including the Gene Naming Committee, Scientific Advisory Board, and members of the *S. pombe* microPublication (Raciti *et al*. 2018) editors board, these rules have been formulated to reflect a consensus.The following rules apply to all types of alleles:

- Alleles within a genotype should be separated by spaces. Consequently, allele names cannot contain spaces.
- Superscripts have been used to indicate resistance or sensitivity in allele names in the past (*can1-1^r^* and *cdc2-5^ts^*), but are deprecated from the early guidelines (Kohli 1987). We encourage moving away from names that cannot be captured in plain text, which are not practical for data mining and storage. For instance, in PomBase superscripts cannot be stored and such alleles would be represented as *can1-1r* and *cdc2-5ts*.
- Allele names must contain only the allowed characters: Latin letters and digits, and the allowed symbols from Tables 2, and 3. Non-ASCII characters are not allowed, except for Δ, since they complicate machine reading.

**Table 2.** Summary of nomenclature for allele gene variant features, as described in the main text.

Variant type		Example	Regex pattern
Wild type		*ase1+*	\+
Knockout/deletion		*ase1Δ*	Δ
Partial deletion	Indicate remaining residues	*ase1(1-35)* *ase1(1-35,40-700)*	\(((?<!\d)\d+-\d+,?)+(?<!,)\)
	Indicate missing residues	*ase1Δ(114-120)* *ase1Δ(114-120,150-200)*	Δ\(((?<!\d)\d+-\d+,?)+(?<!,)\)
Residue insertion		*ase1-P114PVPAL*	−((?<=[,-])[A-Z]+\d+[A-Z*]+,?)+(?<!,)
Substitution or deletion-insertion	Single residues	*ase1-P114A,Q117A* ase1-P114*	
	Consecutive residues	*ase1-PLI114AAA* *ase1-PLI114LAVKK*	
Disruption by insertion		*ase1::kanMX6@(CU329670.1:1878390_1878391ins)*	N/A
Forward genetics, variant unknown		*ase1-35* (must be unique)	N/A
CTD	All repeats mutated	*rpb1-CTD-Y1F*	Ψ?-CTD-((?<=[,-])Ω(\(r\d+-r\d+(−\d+)?\))?,?)+(?<!,)
	Some repeats mutated	*rpb1-CTD-Y1F(r1-r12)*	
	Some repeats deleted	*rpb1-CTD-Δ(r11-r29)*	
	Motifs mutated every two repeats, from repeat 1 to 29.	*rpb1-CTD-Y1F(r1-r29-2)*	
	Combinations of the above	*rpb1-CTD-S5A(r1-r12),Δ(r13-r29)*	
	CTD and non-CTD mutations	*rpb1-N494D-CTD-Δ(r11-r29)*	

The right column includes a regular expression matching characters after the gene symbol. [A-Z*] is used to denote residues and * (stop codon) for simplicity, but regex matching only either DNA or amino acid symbols should be used instead. For clarity, in the CTD regex, “Ψ” is used to indicate a pattern that would match either partial deletion or substitution regex (Δ\(((? <! \d)\d+-\d+,?)+(? <! ,)\)|\(((? <! \d)\d+-\d+,?)+(? <! ,)\)|-((? <=[, -])[A-Z]+\d+[A-Z*]+,?)+(? <! ,)), and “Ω” is used to indicate a pattern that would match either a partial deletion using Δ syntax, a substitution or a deletion (Δ\(((? <! \d)\d+-\d+,?)+(? <! ,)\)|([A-Z]+\d+[A-Z*]+)|Δ). See the GitHub repository (Ramirez 2023) for more details.

**Table 3.** Summary of nomenclature for nonvariant features, as described in the main text.

Character	Meaning	Example
?	The allele present in the locus is unknown, *h?* denotes unknown mating type	*h? ura4?*
+	Wild-type allele (optional except in diploid genotypes and insertion locus)	*ura4+::ase1-GFP* *ura4+/ura4-294*
/	Separator for diploid loci	
::	Indicate locus of insertion	*ase1Δ::kanMX6* *ura4+::ase1-GFP:ura4-294*
.	Indicate a component of a gene outside of the gene product (promoter, terminator, UTR or intron)	*CDS.prp22:natMX6:3UTR.prp22* *ura4+::P.act1-GFP-ase1-T.ase1*
-	Nonfunctional allele which is unknown	*ura4-*
	Separator for subsequent *functionally linked* allele components	*P3nmt1-ase1-GFP-T.nmt1:kanMX6* *ase1Δ::ase1-mal3:kanMX6* *ase1Δ::ase1(1-130)-mal3(4-190):kanMX6*
:	Separator for subsequent *functionally unlinked* allele components	*ura4+::ase1-GFP:ura4-294* *kanMX6:P3nmt1-ase1-GFP* *ura4+::P.ase1-ase1-GFP-T.ase1:P.atb2-mCherry-atb2-T.atb2:kanMX6* *ura4+::loxP:ase1-GFP:loxP:kanMX6*
@	Genome location for insertion or substitution	*ura4::kanMX6@(CU329670.1:3571861_3571862ins)* *P3nmt1-ase1-GFP-T.nmt1:kanMX6@(CU329670.1:1886500_1886750delins)*
\	Prefix indicating the provenance of the sequence (foreign species or plasmid for clarification).	*ura4+::Scer\ACT1-GFP* *ase1-pXYZ\meGFP:kanMX6* *ase1-SV40\NLS:kanMX6* *GFP-ase1-IAA17 ura4+::Atal\TIR1*
( )	Residues of the linker sequence	*ase1-(PGAGAGAGS)-GFP*
	Grouping terms for clarity	*kanMX6:P3nmt1-(ase1-P114-VPAL)-GST*
[ ]	Replicative plasmid. Plasmid name, optionally followed by relevant elements separated by space	*ura4 [pXYZ]* *[pXYZ pREP3\P3nmt1-mCherry-ase1-pREP3\T.nmt1:LEU2]*

## Gene variant features

The nomenclature rules summarized in Table 2 can be used to describe the variants in the allele name:

1. To name the wild-type allele, use the gene symbol followed by a plus sign, *ase1+*. This is only compulsory in diploid genotypes and to indicate insertions at loci where the wild-type allele is still present (see next section).
2. To denote a knockout or gene deletion, use the gene symbol followed by Δ. For example, *ase1Δ*.
3. In allele names of protein-coding genes affecting the gene product, variants should be described at the protein level, referring to amino acid residues, with index 1 corresponding to the translation initiation methionine (except for histones, see “Further guidelines”). For genes with multiple transcripts, see rule 10.
4. In allele names of RNA-coding genes affecting the gene product, variants should be described at the transcript level, with index 1 corresponding to the first transcribed nucleotide. When modifications affect a Uracil nucleotide in the RNA, use T instead of U.
5. For modifications of regions outside of the gene product, we recommend using an allele variant short name (see next section).
6. To denote a partial deletion, use the gene symbol followed by parentheses with the range of retained residues or a Δ followed by parentheses with the range of deleted residues. *ase1(1-113)* and *ase1Δ(114-731)* describe identical alleles, since Ase1 has 731 amino acids. Commas can be used to separate subsequent deletions or retained regions: *ase1Δ(20-114,530-731)* and *ase1(1-19,115-529)* are also identical. We recommend using the Δ notation whenever possible as it emphasizes the modification with respect to the wild-type.
7. Insertions, substitutions, and deletion–insertions should be described with the same syntax. Use the gene symbol, a hyphen, the consecutive replaced residues, the index of the first replaced residue, and the replacing residues.
  - Substitution of a single residue: ase1-P114A, ase1-P114* (use asterisk, “*”, for stop codon).
  - Substitution of consecutive residues: *ase1-PLI114AAA* (P114, L115, and I116 replaced by 3 alanines).
  - Deletion–insertion: *ase1-PLI114VPAAL*, (P114, L115, and I116 replaced by the sequence *VPAAL*).
  - Insertion: *ase1-P114PVAL* (insertion of VAL after P114, to denote the insertion use the same residue before and after the index).
  - The above mutations can be concatenated with commas (without spaces), and combined with a partial deletion: *ase1Δ(20-30)-P114A,E126A*. Indexes should refer to residues in the reference sequence and not the sequence after removal or insertion of residues.
8. To denote disruption of a gene by insertion (cassette interrupting the open reading frame (ORF), for example in a transposon screen (Grech *et al*. 2019)), use the gene symbol, double colons, disruption cassette, @ character, and insertion coordinates in the variant nomenclature format (den Dunnen *et al*. 2016): *ase1::kanMX6@(CU329670.1:1878390_1878391ins)*. For details on @ syntax see “Special characters”.
9. To denote mutations that occur on CTD repeats use the gene symbol, hyphen, “CTD”, hyphen (*rpb1-CTD-*), then:
  - For variants that affect all repeats, append the variant syntax as described above, e.g. *rpb1-CTD-Y1F*.
  - For variants that affect certain repeats, indicate the repeats where the changes were made using “r” followed by the repeat number in parenthesis. Repeats can be specified as a list of comma-separated values *rpb1-CTD-Y1F(r1,r2)*, or a range in the form start-stop-step where the default step is one. For example, *rpb1-CTD-Δ(r11-r29)* to indicate repeats 11 to 29 are deleted, *rpb1-CTD-Y1F(r1-r29-2)* to indicate that mutation Y1F is present every 2 repeats, from repeat 1 to 29.
  - Several mutations can be combined using the above syntax, one after the other, comma-separated e.g. *rpb1-CTD-S5A(r1-r12),Δ(r13-r29)*.
  - Mutations in the CTD and non-CTD part of the protein can be combined, e.g. *rpb1-N494D-CTD-Y1F(r1-r12)* to indicate mutation of the single residue N494 outside the CTD and Y1F mutations in all repeats. Because the comma is also used to separate multiple modifications in the non-CTD part of the protein, multiple modifications in the CTD should always go last. For example, in "*rpb1-S2A,N494D-CTD-Y1F,S5A*" "S2A" and "N494D" refer to the non-CTD part, and "Y1F" and "S2A" refer to the CTD part.
10. For alleles of genes with multiple transcripts, we consider two cases:
  - If the affected residues exist in the primary transcript, the primary transcript sequence should be used as a reference, and the allele name should start with the gene symbol, as described above. The primary transcript is the one whose identifier ends with “.1” and is shown by default in gene pages in PomBase.
  - If the affected residues exist only on a transcript other than the primary, or cDNA is being used so that no other transcript can be produced, the sequence of the concerned transcript should be used as a reference. In that case, the allele name should start with the gene symbol followed by a period and the transcript number, e.g. “*zas1.2-R89A*”.

To help with machine readability, we provide regular expressions matching the example patterns in a publicly accessible GitHub repository (Ramirez 2023), summarized in Table 2.

## Allele variant short names

Alleles isolated from forward genetic experiments (e.g. suppressor screen) where the lesion is known should use the above syntax describing the mutations. However, if the mutations are unknown, a short name can be used. The short name must be unique, starting with the gene symbol followed by a hyphen and a combination of letters and/or digits.

Short names are also recommended in cases where an allele capturing all variant information would be too long (e.g. *rap1-32E*, where 32 residues are mutated to glutamate (Amelina *et al*. 2015)) or for alleles affecting regions outside of the gene product, including introns. The variant name capturing all modifications should be included at least in the methods section, and stored in the relevant model organism database at curation time.

Periods (“.”) have been used interchangeably in place of hyphens (“-”) in allele short names (e.g. *cut12.s11*). Although existing alleles will not be renamed, new allele short names should observe hyphens as separators and periods should be used in certain allele names of genes with multiple transcripts (see previous section), or as described in Table 3. Underscores are not allowed in allele variant short names and will be changed to hyphens in existing names with the original names retained as synonyms.

## Special characters and nonvariant features

**Fig. 1.**
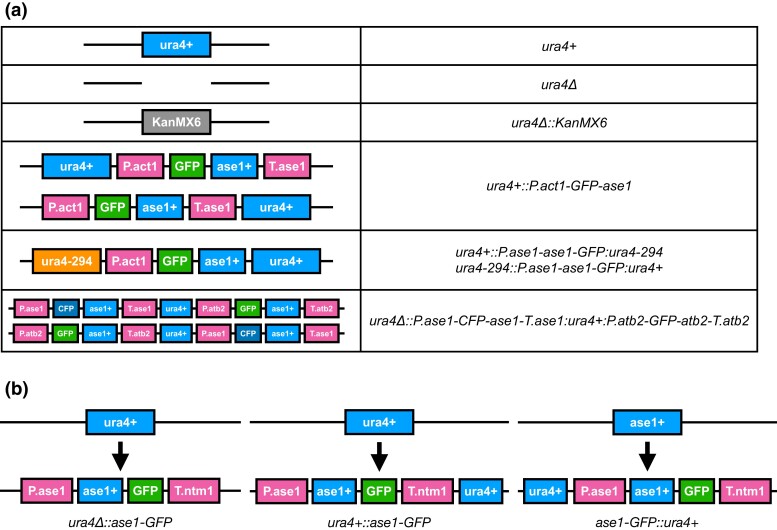
Graphical representation of representative alleles and their corresponding nomenclature. a) Alleles present in the *ura4* locus and their corresponding names. In order: (1) The wild-type locus; (2) A deletion of the ORF without replacement (e.g. scar-free CRISPR-Cas9); (3) A deletion allele in which the *ura4* locus was replaced by a KanMX6 cassette. Note that the deletion allele *ura4Δ* is used both to indicate the locus of insertion and the fact that the *ura4* ORF is missing; (4) This allele name can represent the insertion of *P.act1-GFP-ase1* at either side of the *ura4* locus since the transcription orientation is not captured in the allele name; (5) For the same reason, a construct containing 2 flanking *ura4* alleles can have 2 synonymous names when inserted in the *ura4* locus; (6) Allele names should capture the insertion locus and the relative position of features, rather than how alleles were generated or their genetic meaning. Therefore, in a case where an allele of the insertion locus (*ura4+* allele in the image) is in the middle of the construct, we use *ura4Δ::* to indicate the insertion locus, followed by the construct name, including the *ura4+* allele. b) Special cases in which an allele name contains 2 gene symbols (*ase1* and *ura4*), and one of them is also the locus of insertion. The wild type locus is shown on top, and the resulting alleles and their names at the bottom: (left) If inserted in the *ura4* locus replacing the *ura4* ORF, the allele name starts with “*ura4Δ*::”; (center) If inserted in the *ura4* locus keeping the *ura4 ORF*, the allele name starts with “*ura4+::”*; (right) If inserted in the *ase1* locus, the allele name starts with “*ase1-GFP::*”.

The current *pombe* genetic toolbox is extremely versatile. Rather than capturing every possible type of allele that can be generated and the relationships between allele features, we provide rules and characters to connect allele features in a modular way to describe genotypes (Table 3):

1. Use the “?” character when the allele of a gene is unknown. For example “*h?*” to indicate that the mating type is unknown, or “*ura4?*” when the allele present at the *ura4* locus is unknown (this could be the wild-type allele or any other *ura4* allele).
2. Use the “/” character to separate alleles present in the same diploid locus, e.g. (*ura4+/ura4-294*).
3. Use the “+” character after the gene symbol to indicate the wild-type allele (e.g. *ura4+*) (Kohli 1987). This notation is mandatory for wild-type alleles in diploid genotypes, or insertion loci where the wild-type copy is still present (see rule 4).
4. Use double colon “::” to indicate the insertion locus when needed (see rule 14). The insertion locus should always come first in the allele name. If the insertion replaces the ORF of the locus creating a deletion, indicate so with a “Δ”. For example, use *ura4Δ::kanMX6* for a deletion where the *ura4* ORF has been replaced by the *kanMX6* cassette (Fig. 1a) and *ura4Δ::P3nmt1-GFP-ase1* for a replacement of the *ura4* ORF by *P3nmt1-GFP-ase1*. If the construct contains an allele of the insertion locus, use the allele name instead to indicate the locus. For example, *ura4+::ase1-GFP* for insertion at the *ura4* locus where a wild-type copy of the gene is still present, using the vector pUra4AfeI (Vještica *et al*. 2019, January 1) (Fig. 1b). The original Köhli guidelines propose using only the locus gene symbol to indicate the insertion locus. Instead, our guidelines propose using an allele of the insertion locus, since an allele name like *ura4::ase1-GFP* is ambiguous (Fig. 1b).
5. Use a period “.” to define a subcomponent of the gene structure: *P.act1* (promoter of *act1*), *T.act1* (terminator of *act1*), *CDS.act1* (coding sequence of *act1*), *5UTR.act1*, *3UTR.act1*, *intron1.act1*… This notation can be used to indicate a promoter switch (e.g. *P.act1-ase1*, *ase1* promoter replaced by *act1* promoter) or to describe DAmP (Decreased Abundance by mRNA Perturbation) alleles (Nissen *et al*. 2017), where a resistance marker is introduced in a coding gene after the stop codon, changing the 3′UTR (e.g. *CDS.prp22:natMX6:3UTR.prp22*). There is no precedent for this notation in the literature, and researchers have often used pact1, act1p, act1pr, Pact1 and others to refer to promoters, and similar syntax has been used for terminators. However, these notations pose problems for gene symbols that may not end with a number (genes from other species), and would not support UTRs. We have deliberately excluded the prime symbol from the UTRs to avoid character inconsistencies. For promoters and terminators, we recommend clearly indicating in the methods the range of residues that were cloned upstream of the ATG or downstream of the STOP.
6. Use the hyphen character “-”:
  - Between allele components that are functionally linked, either by being part of the same gene product (*ase1-GFP*) or through regulation (*P.act1-ase1-GFP*). Combined with the variant syntax, this can be used to describe gene fusions (*mal3-ase1*) and chimeras (*ase1(1-130)-mal3(4-190)*).
  - Use “-” after gene symbols to indicate that a locus contains an unknown loss-of-function allele (*ura4-*). We recommend the use of a hyphen (unicode “hyphen-minus” U+002D) over a minus sign (unicode “minus sign” U+2212) as these are often not distinguishable when reading, and U+002D is available in common keyboard layouts.
7. Use single colon “:” between allele components that are adjacent but not functionally linked (selection markers, recombination sites, adjacent products). For example, *ura4Δ::P.ase1-ase1-GFP:P.atb2-mCherry-atb2:kanMX6* represents the replacement of the *ura4* ORF by two fluorescently labeled proteins in tandem followed by a resistance marker.
8. Use the character “@” followed by sequence variant nomenclature in parenthesis to indicate exact insertion coordinates (see (den Dunnen *et al*. 2016)). For example, use *ura4::kanMX6@(CU329670.1:3571861_3571862ins)* for an allele that was generated by the insertion of a transposon containing *kanMX6* between nucleotides 3571861 and 3571862 of Chromosome I. CU329670.1 is the INSDC identifier of chromosome I in genome assembly ASM294v2. Alternatively, a RefSeq identifier (Pruitt *et al*. 2007) can be used. For insertions that disrupt the gene product, as in the example above, only the insertion locus (*ura4::*) should be indicated. The deletion-insertion syntax can also be used (see Table 3).
9. Use the backslash “\” to add a prefix indicating the provenance of an allele component (foreign species or plasmid). This is compulsory for genes from other species, e.g. *Scer\ACT1* to refer to the ACT1 gene from *S. cerevisiae*. Additionally, it can be helpful to understand what exactly the allele contains, e.g. *SV40\NLS* to indicate the NLS from Simian Virus 40, or *pREP3\P3nmt1* to indicate that the P3nmt1 promoter comes from the plasmid pREP3. This notation is used by FlyBase (Thurmond *et al*. 2018).
10. Use square brackets “[ ]” to indicate the name of an extrachromosomal replicating plasmid present in a strain. For instance, *h- ura4-D18 [pXYZ]* to describe a strain containing the plasmid pXYZ. Optionally, after the plasmid name, add a space and indicate relevant elements of the plasmid, using the same syntax described above, e.g. *[pXYZ pREP1\P3nmt1-mCherry-ase1-pREP1\T.nmt1:LEU2]* indicates that pXYZ is a pREP1-derived plasmid driving the expression of *mCherry-ase1*.
11. Use parenthesis “( )”:
  - To indicate residues used as a linker between allele features, e.g. *ase1-(PGAGAGAGS)-GFP*. If the linker is too long, “linker” can be used instead: *ase1-linker-GFP*.
  - To optionally group a substring within the allele name to avoid confusion. For example, writing *kanMX6:P3nmt1-(ase1-P114PVAL)-GST* helps separate the variant component of the allele (*ase1-P114PVAL*, an insertion of amino acids VAL after residue P114), from the GST tag, which could be misinterpreted as a group of three amino acids.
12. Elements should be listed in the order they appear in the sequence, but their transcription orientation need not be captured (*ura4+::P.act1-GFP-ase1* in Fig. 1a).
13. If a construct contains two variant features derived from the insertion locus, either of them can be used before the :: to indicate the insertion locus. For instance, when describing an allele generated using pJK210 (Keeney and Boeke 1994), *ura4+::P.ase1-ase1-GFP:ura4-294*, and *ura4-294::P.ase1-ase1-GFP:ura4+* are synonymous (Fig. 1a).
14. When an allele name only contains one gene symbol from the host species, it is assumed that the allele is in the endogenous locus. For example, *mCherry-atb2:kanMX6* is identical to *atb2Δ::mCherry-atb2:kanMX6*. This is not valid in the case where *ura4+* or other *pombe* gene is used as a selection marker, in which case the allele should be called *ase1-GFP::ura4+* if it is in the *ase1* locus, and *ura4+::ase1-GFP* if it is in the *ura4* locus (Fig. 1b).
15. It is compulsory to indicate the locus of insertion for gene fusions and chimeras, to clarify whether the wild-type allele of either of the fused genes is still present.

## Further guidelines and exceptions

1. Genotypes in strain lists should capture the insertion locus and the relative position of features, rather than how alleles were generated or their genetic meaning. For example, if two constructs were independently inserted before and after the *ura4* locus, the way to describe the genotype would be *ura4Δ::P.ase1-CFP-ase1-T.ase1:ura4+:P.atb2-GFP-atb2-T.atb2*. Note how the name contains *ura4Δ*, even if the *ura4+* wild-type copy is present (Fig. 1a).
2. Strain lists or any other list of biological resources in the supplementary material should not be published as a pdf. This greatly limits machine readability, especially for characters such as hyphens, Greek letters, etc.
3. We recommend including all maps of plasmids used for allele generation and transformation in the supplementary material in GenBank or equivalent format. Papers should also include a list of the primers used for cloning and describe cloning strategies in sufficient detail.
4. Although allele names in the main text of publications are generally short (e.g. locus of insertion and resistance markers are normally not mentioned), there are some exceptions, particularly for chimeras. We recommend declaring synonyms in the text along with the full description so that text mining can detect them in the future, for example: “We generated two chimeras, *klp6(1-410)-klp5(405-883)*, and *klp5(1-404)-klp6(411-784)*, referred to as *klp6N-klp5C* and *klp5N-klp6C*” (Unsworth *et al*. 2008). For strain lists, we recommend being as explicit as possible and avoiding short names.
5. Across organisms, histone amino acid positions are described based on numbering after the removal of the initiator methionine. This convention should be used in allele names.
6. We discourage the use of truncation allele names ending in ΔN, ΔC, ΔNterm, ΔCterm, and similar names, for example, *gar2ΔNterm*. This can lead to clashes with truncations from other publications removing a different set of amino acids in the same protein.
7. When a gene from another species is mentioned in the main text, we recommend stating this clearly so that Natural Language Processing can associate the gene symbol with the species. For example, use “*ase1*, the homolog of human PRC1” instead of “*ase1*, the homolog of PRC1”.

## Harmonization of commonly used allele components

Derivatives of the *S. pombe nmt1* promoter are commonly used to drive gene expression. The most commonly used constructs have been derived from pREP3, pREP41, and pREP81 (Maundrell 1990; Basi *et al*. 1993) and contain the *nmt1* promoter with minor mutations (pREP3), and attenuated versions in which the TATA box was truncated (pREP41, pREP81). In published strain lists, these promoters have been referred to in different ways (e.g. P3-nmt1, P3Xnmt1, P3nmt1, Pnmt1 for the promoter in pREP3). We recommend following the nomenclature from (Bähler *et al*. 1998), referring to them as P3nmt1, P41nmt1, and P81nmt1.

Chemical resistance markers used in *S. pombe* are often derived from *kanMX* constructs originating from plasmid pAG224 (Wach *et al*. 1994). pAG224 contains the kanamycin resistance gene from *E. coli* transposon Tn903 (Oka *et al*. 1981) flanked by the promoter and terminator from the Transcription Elongation Factor (TEF) gene of *Ashbya gossypii* (Steiner and Philippsen 1994). The kanamycin resistance cassette was subsequently substituted by constructs conferring resistance to nourseothricin (NAT, from *S. noursei* gene *nat1*), hygromycin B (from *K. pneumoniae* gene *hph*) and phleomycin (from *S. hindustanus* gene *ble*) (Hentges *et al*. 2005). If *kanMX*-derived markers have been used, we recommend referring to them as *natMX6*, *hphMX6*, *bleMX6*, and *kanMX6*, rather than *nat^r^, hyg^r^, ble^r^,* and *kan^r^*.

## Conclusion

These revised guidelines for noncoding RNA gene symbols and updated allele/genotype nomenclature will improve consistency, facilitate machine-readability, and aid automated entity recognition in fission yeast publications and datasets. This is increasingly important as knowledge bases and journals incorporate Artificial Intelligence and Natural Language Processing into their curation, some examples being pipelines to triage publications for curation (Poux *et al*. 2017) or identification of biological entities in the text of articles (Müller *et al*. 2018; Raciti *et al*. 2018). As tools leveraging Large Language Models become more accessible, we anticipate that funders will increasingly support projects to automate parts of the curation process and this will require data in standardized formats and training sets defined by experts (Wood *et al*. 2022). In particular, named entity recognition tools require precompiled lexica of existing entities and defined patterns for the description of novel genetic features. Adhering to allele nomenclature will benefit journals like microPublication (Raciti *et al*. 2018), which link genetic entities in publication text to relevant knowledge bases. We were partly motivated in this work by inconsistencies in the automated markup of genetic constructs by our microPublications partners.

Adopting nomenclature standards will enable automation, reducing routine data input tasks and freeing up curators to focus on knowledge extraction and standardization, making causal connections, and quality control, which require domain expertise. This automation step will also motivate curation by authors, which in PomBase has matched or exceeded professional curation in content creation since 2021 (Lock *et al*. 2020). We aim to automate the import of genetic entities from publications into the curation tool Canto (Rutherford *et al*. 2014) to improve the curator experience and further lower the friction for authors to engage in curation (Lock *et al*. 2020).

In summary, our updated guidelines promote consistency, clarity, and FAIRness for genetic data sharing and interpretation that will facilitate machine readability and automation of the curation process.

## Data Availability

The full list of alleles from PomBase and JaponicusDB can be downloaded at the Phenotype Annotations page: https://www.pombase.org/downloads/phenotype-annotations; https://www.japonicusdb.org/downloads/phenotype-annotations. The code with regular expression mentioned in the text is in GitHub (https://github.com/pombase/allele_gene_variant_features_regex). The version used for this manuscript is deposited in Zenodo (https://doi.org/10.5281/zenodo.8139931).
